# Personalized Medicine to Improve Treatment of Dopa-Responsive Dystonia—A Focus on Tyrosine Hydroxylase Deficiency

**DOI:** 10.3390/jpm11111186

**Published:** 2021-11-12

**Authors:** Gyrid Nygaard, Peter D. Szigetvari, Ann Kari Grindheim, Peter Ruoff, Aurora Martinez, Jan Haavik, Rune Kleppe, Marte I. Flydal

**Affiliations:** 1 Division of Psychiatry, Haukeland University Hospital, 5021 Bergen, Norway; gyrid.nygard@uib.no (G.N.); jan.haavik@uib.no (J.H.); 2 Department of Biomedicine, University of Bergen, 5009 Bergen, Norway; peter.szigetvari@uib.no (P.D.S.); Ann.grindheim@uib.no (A.K.G.); aurora.martinez@uib.no (A.M.); 3 Department of Chemistry, Bioscience and Environmental Engineering, University of Stavanger, 4021 Stavanger, Norway; peter.ruoff@uis.no; 4 Norwegian Centre for Maritime- and Diving Medicine, Department of Occupational Medicine, Haukeland University Hospital, 5021 Bergen, Norway

**Keywords:** tyrosine hydroxylase, dopamine, dopa-responsive dystonia, dystonia, neurometabolic disorders, personalized medicine, computational modeling, tyrosine hydroxylase deficiency, L-DOPA

## Abstract

Dopa-responsive dystonia (DRD) is a rare movement disorder associated with defective dopamine synthesis. This impairment may be due to the fact of a deficiency in GTP cyclohydrolase I (GTPCHI, *GCH1* gene), sepiapterin reductase (SR), tyrosine hydroxylase (TH), or 6-pyruvoyl tetrahydrobiopterin synthase (PTPS) enzyme functions. Mutations in *GCH1* are most frequent, whereas fewer cases have been reported for individual SR-, PTP synthase-, and TH deficiencies. Although termed DRD, a subset of patients responds poorly to L-DOPA. As this is regularly observed in severe cases of TH deficiency (THD), there is an urgent demand for more adequate or personalized treatment options. TH is a key enzyme that catalyzes the rate-limiting step in catecholamine biosynthesis, and THD patients often present with complex and variable phenotypes, which results in frequent misdiagnosis and lack of appropriate treatment. In this expert opinion review, we focus on THD pathophysiology and ongoing efforts to develop novel therapeutics for this rare disorder. We also describe how different modeling approaches can be used to improve genotype to phenotype predictions and to develop in silico testing of treatment strategies. We further discuss the current status of mathematical modeling of catecholamine synthesis and how such models can be used together with biochemical data to improve treatment of DRD patients.

## 1. Introduction

**Figure 1:**
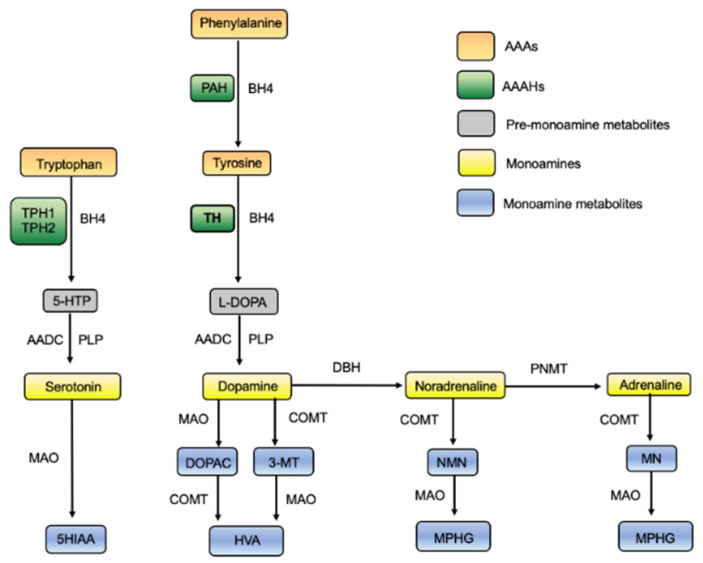
Simplified overview of the biosynthesis and catabolism of serotonin and the catecholamines. Note that different parts of these processes take place in different tissues (see text for details). Abbreviations: 3-MT, 3-methoxytyramine; 5-HTP, 5-hydroxytryptophan; 5HIAA, 5-hydroxyindolacetic acid; AAAHs, aromatic amino acid hydroxylases; AAAs, aromatic amino acids; AADC, aromatic acid decarboxylase; BH4, (6R)-L-erythro-5,6,7,8-tetrahydrobiopterin; COMT, catechol-O-methyltransferase; DBH, dopamine beta-hydroxylase; DOPAC, 3,4-dihydroxyphenylacetic acid; HVA, homovanillic acid; L-DOPA, L-3,4-dihydroxyphenylalanine; MN, metanephrine; MAO, monoamine oxidase; MPHG, 3-methoxy-4-hydroxyphenylethylene glycol; NMN, normetanephrine; PAH, phenylalanine hydroxylase; PLP, pyridoxal phosphate; PNMT, phenylethanolamine N-methyltransferase; TH, tyrosine hydroxylase; TPH, tryptophan hydroxylase.

Dopa-responsive dystonia (DRD) is a group of disorders that typically present with childhood onset diurnally fluctuating limb dystonia, thus also referred to as hereditary progressive dystonia with marked diurnal fluctuation [1,2]. The conditions that manifest as DRD are clinically heterogenous and non-motor symptoms with neuropsychiatric features, for example, depression, may also be present in some cases [3]. DRD is rare, with an estimated prevalence of 0.5–1 per million worldwide [4,5], and it is mainly caused by abnormalities affecting the biosynthesis of catecholamines (CAs). Monoamine neurotransmitters, such as serotonin, dopamine, and other CAs, are synthesized in reaction pathways involving the tetrahydrobiopterin (BH4)-dependent aromatic amino acid hydroxylases (AAAHs) (Figure 1). DRD results from deficient 3,4-dihydroxyphenylalanine (L-DOPA) synthesis, either through insufficient BH4 production or from defective tyrosine hydroxylase (TH; EC 1.14.16.2; tyrosine 3-monooxygenase), the enzyme that catalyzes the conversion of tyrosine (Tyr) to L-DOPA, the precursor of CAs (Figure 1). The most commonly reported causes of DRD are mutations in the genes encoding enzymes of the BH4 biosynthetic pathway. Segawa disease, caused by autosomal dominant GTP cyclohydrolase I (GTPCHI) deficiency from mutations in the *GCHI* gene [6], is the most frequent condition that manifests as DRD, whereas fewer cases are described for deficiencies in sepiapterin reductase (SR) [7], 6-pyruvoyl tetrahydrobiopterin synthase (PTPS) [8], and TH [2,9]. It is important to note that with the increased use of novel diagnostic tools, such as next-generation sequencing [10], the group of genes associated with DRD is currently expanding, with cases also being reported for mutations in genes including ataxia telangiectasia [4] and nuclear receptor 4A2 [11]. Although most DRD patients have a sustained beneficial response to levodopa (L-DOPA), which is used to restore dopamine levels in disorders with deficient dopamine synthesis [12], some patients respond poorly or not at all. As this is often the situation for severe cases of TH deficiency (THD; OMIM *191290), a rare neurometabolic disorder inherited in an autosomal recessive manner, we focused more intensively on this patient group. A combination of an improved selection of treatment options and a personalized medicine (PM) approach is likely to help these patients. In this review, we focused on THD pathophysiology and ongoing efforts to develop novel therapeutics for this disorder and describe how different modeling approaches can be used to improve genotype to phenotype predictions and for in silico testing of treatment strategies.

### 1.1. The Role of TH

The hydroxylation of Tyr to L-DOPA catalyzed by TH is the rate-limiting step in the biosynthesis of the CA neurotransmitters and the hormones dopamine, noradrenaline, and adrenaline [13,14] (Figure 1). CAs are ancient signaling molecules with key roles in multiple physiological functions, and their levels are therefore tightly regulated. Dopamine is involved in motor control, cognition, memory, and reward, and is a precursor of adrenaline and noradrenaline, which also act as neurotransmitters and hormones, regulate attention and helping regulate cardiovascular function and metabolic activity [15]. In addition to their canonical effect on G-protein coupled receptors, dopamine and other CAs can bind to target proteins, including TH, via tight charge–transfer interactions with the active site iron [16,17]. Such diverse mechanisms may contribute to the multifaceted and long-lasting effects of these molecules.

### 1.2. The Clinical Manifestations of THD

In patients with THD, the enzymatic activity of TH is compromised, mainly due to the loss-of-function mutations in the *TH* gene (chromosome 11p15.5) [13,18,19]. The resulting cerebral CA deficiency causes a complex pathophysiology involving different brain areas and functions and presents with considerable heterogeneity in phenotype amongst the small group of patients that have been diagnosed with THD so far. This probably explains why the first reported *TH* mutation in patients was detected as late as in 1995 [19].

In 2010, the clinical phenotypes caused by pathogenic variants in the *TH* gene were suggested to mainly fit into two main groups: type A and type B [20]. Type A is characterized as a progressive, hypokinetic-rigid syndrome with dystonia and onset during infancy or childhood, while Type B presents as a complex encephalopathy with onset in the neonatal period or early infancy. In practice, the observed THD phenotypes do, however, fit along a spectrum with overlap of both clinical features and *TH* mutations between the two groups [20]. The recent International Working Group on Neurotransmitter-Related Disorders registry study employed the first standardized deep phenotyping approach with 44 THD patients and concluded that the type A/B classification is not justified and proposed to abandon this classification entirely [21]. Thus, while a categorization based on symptoms may assist clinicians towards achieving more personalized care for different patient cohorts, in the case of THD, such efforts have yielded limited success to date. Due to the low number of THD patients and overlapping symptoms with other disorders, early diagnosis and treatment remain challenging.

### 1.3. Diagnosing THD

Underdiagnosis is a major issue with THD and differential diagnosis include epilepsy [22], cerebral palsy [22], and vitamin B12-deficiency related infantile tremor syndrome [23] among others. It is important to reach a correct diagnosis at an early stage, as many patients are responsive to treatment. The clinical diagnosis of THD is based on neurotransmitter metabolite analysis in CSF from lumbar puncture, followed by mutation analysis of the *TH* gene [24], with the latter being the only confirmative option. To our knowledge, only two instances of prenatal diagnosis have been reported, both performed on genomic DNA extracted from the chorion-villus at 11 weeks of gestation [25,26]. In the second pregnancy of a Caucasian family with a severely affected daughter heterozygous for the R328W and T399M mutations (residue numbers based on the human TH type 4, 528 residues; NP_954986.2; P07101-1), prenatal diagnosis revealed THD in the fetus and was followed by abortion. The third pregnancy resulted in spontaneous abortion at 16 weeks and mutational analysis of the fetus showed the THD genotype in this case as well (see below) [25]. On the contrary, carrier status was confirmed prenatally in a Chinese fetus with the common mutation R233H and the novel mutation R476S [26]. The first pregnancy led to a girl with severe THD symptoms from the first year but with a dramatic improvement when treated with L-DOPA, suggesting that early treatment in affected infants may prevent the development of symptoms. In cases with no previous family history of THD (which applies to the majority), the disease is generally first indicated by the occurrence of central CA deficiency, where the CSF presents normal levels of 5-hydroxyindolacetic acid (5HIAA), low levels of homovanillic acid (HVA) and 3-methoxy-4-hydroxyphenylethylene glycol (MHPG), and a low HVA:5-HIAA ratio (Figure 1) [20]. The complete selection of tests usually depends on the clinical presentation and the phenotypic complexity of the individual patients and has been reviewed elsewhere [12].

Patients diagnosed with THD are mainly treated with L-DOPA, still, a considerable percentage of affected individuals—primarily those more severely hit—respond poorly. Even within the responsive group, L-DOPA treatment can be associated with side effects and loss of clinical efficacy over time [20,27]. Thus, there is an unmet clinical need for alternative therapies in THD. Of note, reduced TH activity and dopamine levels are also associated with Parkinson’s disorder (PD) [28,29], dystonia-related movement disorders [30], and Alzheimer’s disease [31,32]. Consequently, efforts to develop new therapeutics based on restoring TH function and activity also have the potential to benefit patients suffering from these disorders.

### 1.4. THD—An Orphan with Challenges Common for Rare Disorders

THD is classified as a rare or orphan disorder both in the US (defined as <200,000 patients nationwide) and elsewhere in the world (defined with a prevalence ranging from <1:2000–<1:50,000) [33,34,35] and have some challenges in common with the majority of the other rare disorders classified to date. For example, approximately 80% of rare disorders have an underlying genetic basis and about 75% affect children [34]. In addition, the low number of patients, limited understanding of disease pathogenesis, and phenotypic variability typically hinder efficient treatment comparisons and high-power statistical analyses when studying these disorders [33,36,37,38]. These challenges have made meeting regulatory requirements for drug development and approval difficult [38]. Moreover, the high monetary costs and assumed limited earning potential associated with drug development for small patient groups have traditionally caused a lack of interest from the pharmaceutical industry. Therefore, rare disorders are presented with a unique set of challenges, as the cumulative disease burden and societal costs are substantial, but for the individual disorder that only affects very few patients, drug development is difficult and associated with high financial costs. As a result, most rare disorders, including THD, currently represent an unmet medical need worldwide. However, the advent of personalized medicine (PM) in combination with new “omics technologies” over the recent years have led to a better molecular characterization of disease, and a new understanding of the general complexity of disease has emerged [39]. Some argue that based on the molecular interindividual differences revealed by the “omics technologies”, PM applies a novel understanding of disease where all become rare due to the uniqueness of each patient [39]. This ongoing paradigm shift in the understanding of disease will probably have major implications for the development and regulatory requirements for novel therapeutics and will likely benefit patients suffering from disorders, such as THD, that historically have been classified as “rare”.

### 1.5. Tyrosine Hydroxylase

#### 1.5.1. The Aromatic Amino Acid Hydroxylases

Together with phenylalanine hydroxylase (PAH; MIM #612349) and tryptophan hydroxylase 1 and 2 (TPH1; MIM #191060, TPH2; MIM #607478), TH is part of the small family of structurally and functionally related enzymes called the AAAHs (Figure 1). These enzymes all require a catalytic non-heme ferrous iron, BH4 as cofactor, and molecular oxygen as an additional substrate [40]. While TPH1 and TPH2 are involved in the production of the indolamines serotonin and melatonin, PAH and TH are both part of the metabolic pathway responsible for producing the three CAs: dopamine, noradrenaline, and adrenaline.

In CA synthesis (Figure 1), Tyr is the initial substrate for TH and can be extracted from food through metabolic processes in the small intestine or be produced from hydroxylation of phenylalanine by PAH in the liver when the intake of Tyr in the diet is low [41]. Tyr from either the small intestine or the liver is then absorbed into circulation and crosses the blood–brain barrier (BBB) before entering the dopaminergic neurons, where TH can catalyze the hydroxylation of Tyr to L-DOPA by the insertion of a single atom from molecular oxygen onto the Tyr aromatic ring. The remaining oxygen atom is reduced to water with BH4 acting as an electron donor [42,43]. L-DOPA is then converted to dopamine by aromatic acid decarboxylase (AADC), and dopamine may, in turn, act as a precursor for the production of noradrenaline and adrenaline (see Figure 1 for details).

PAH is the most studied AAAH, as its deficiency causes phenylketonuria (PKU), the most common inborn error of metabolism (see [44] for a recent review). For this reason, PKU has become a model disease for rare inherited metabolic diseases with >1200 registered mutations in PAH (www.biopku.org, accessed on 10 September 2021). The AAAHs show a high degree of amino acid similarity in their central catalytic domain, where most of the missense variants are reported. This region is also where most high-resolution structural data are available (see below). Thus, based on the high sequence similarity between TH and PAH in the catalytic domain, there should be much to learn from investigations of the correlation between the mutation position and the effect on activity and stability in both PAH and TH missense variants. So far, most PAH mutations are associated with decreased stability and misfolding, lower intracellular half-life, and decreased protein levels in cells [45,46,47]. Formation of aggregates in prokaryote expression systems and in vitro assays is also a feature of several PKU-associated PAH mutations [48], which may be related to the high propensity of even wild-type (WT) PAH to aggregate upon partial denaturation, a feature which is much less pronounced in TH [49]. The formation of amyloid-like aggregates for the PAH-R261Q mutant has also recently been shown [48].

In addition to providing Tyr, the PAH-catalyzed reaction is the rate-limiting step in the degradation of phenylalanine, preventing neurotoxic accumulation of this amino acid. It is important to note that the AAAs and the other large neutral amino acids (LNAAs) rely on the same large neutral amino acid transporter (LAT1) in the brain endothelium and nerve cells. Hence, the LNAAs are mutual competitive inhibitors for import, and metabolic disturbances where the circulating level of one LNAA increases will therefore inhibit transport of the others into the brain. Such imbalances can become critical and severely disturb brain functions. Under normal physiological conditions there are several regulatory processes that enforce a narrow range of circulating AAA levels as well as mechanisms that control the enzymatic activity of the AAAHs (discussed in detail for TH below).

#### 1.5.2. TH Location and Isoforms

In the human central nervous system (CNS), TH is mainly expressed in dopaminergic neurons in the ventral tegmental area (VTA), which projects to the *nucleus accumbens* and the prefrontal cortex and is involved in motivation, addiction, and reward, and in dopaminergic neurons in the *pars compacta* compartment of the neighboring *substantia nigra* which projects to the striatum and plays a central role in motor control. TH is also found in noradrenergic neurons of the *locus coeruleus*, a region in the brainstem involved in stress and panic responses as well as in small groups of adrenaline neurons in the brain stem [50]. Outside the CNS, TH is found in the sympathetic neurons and in the chromaffin cells of the adrenal medulla [51,52,53], and it has also been detected in the pancreas, stomach, and lymphoid tissues [54,55,56,57,58,59].

Most human genes generate multiple mRNA isoforms, but alternative isoforms are especially common in the CNS where they contribute in important ways to a wide range of neural functions [60]. Interestingly, brain isoforms tend to be more commonly found in higher vertebrates compared to other species [61,62]. This is also the case for TH; while most mammals have a single form of TH, the higher primates have two and humans may be the only species with four TH isoforms [63,64,65].

The human isoforms (hTH1-4) are produced by alternative pre-mRNA splicing from a single gene [66,67]. The *TH* gene is located on chromosome 11p15.5 and contains 14 exons with an open reading frame of 1491 base pairs encoding for 497 amino acids [51]. The alternative splicing is from the use of two donor sites in exon 1 and inclusion/exclusion of exon 2, and the resulting isoforms differ by the combinatorial insertion of 4 and 27 amino acid residues [18,65,67,68]. The hTH1 isoform has no insertions and is the shortest form with 497 amino acids and is also the isoform studied the most in vitro as a recombinant enzyme [18,69,70]. The hTH2 and hTH3 isoforms have insertions of 4 or 27 amino acid N-terminals of the positions corresponding to the Ser31 residue of hTH1, respectively [65,68]. The last and longest form, hTH4, has an insertion of all 31 (4 + 27) amino acids and is the most widely used in the literature when referring to positions of missense and nonsense mutations in the *TH* gene [18,65,68]. In the CNS, the hTH1 and hTH2 isoforms are the most abundant and together account for over 90% of the expressed TH protein. Both the hTH3 and hTH4 isoforms are present in very low levels in brain tissues, with enrichment in the nerve terminal field regions (caudate and putamen) over the *substantia nigra* [71].

Different regulatory properties have been detected for the four human isoforms. Several studies are currently ongoing to elucidate more about how their roles differ under normal and pathological conditions (described more in detail below) [18,71,72,73].

#### 1.5.3. TH Structure and THD Mutations

**Figure 2:**
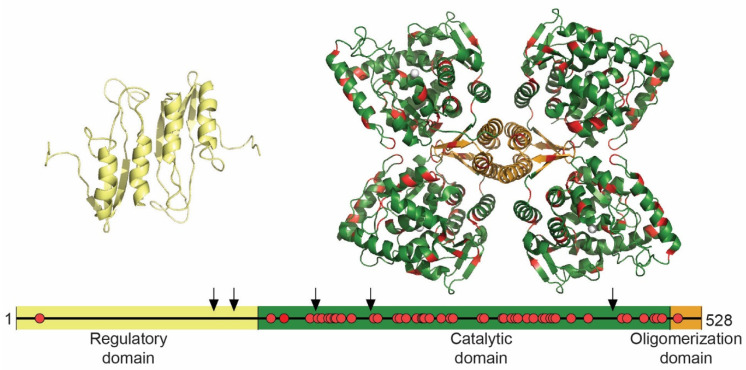
Available TH structures and position of THD mutations. The isolated RD dimer from rat (PDBID 2MDA) [77] (yellow) and the human TH tetramer containing CD (green) and OD (orange) (PDB ID 2XSN). The position of residues with missense mutations registered in PNDdb are shown in red on the structure and on the schematic of the 528 amino acids long TH4. Arrows point to position of nonsense mutations.

**Figure 3:**
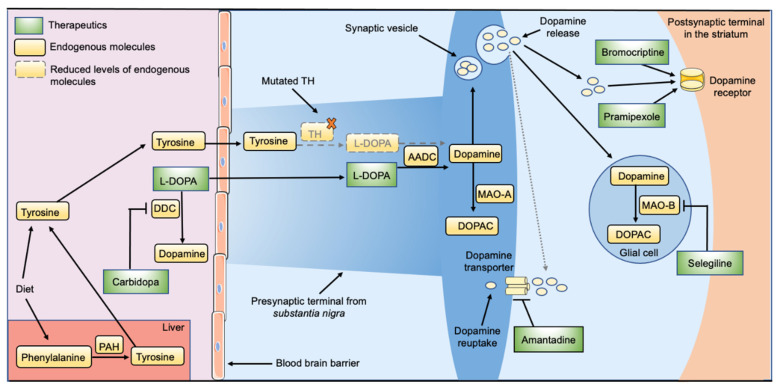
Current therapeutics for treating THD. THD patients are mainly given L-DOPA in combination with an AADC inhibitor (carbidopa) that prevents its peripheral metabolism. Dopamine agonists and inhibitors of dopamine metabolism are also used for some patients, normally in conjunction with L-DOPA/carbidopa. See text for details. Abbreviations: AADC, aromatic acid decarboxylase; DOPAC, 3,4-dihydroxyphenylacetic acid; L-DOPA, L-3,4-dihydroxyphenylalanine; MAO-A, monoamine oxidase A; MAO-B, monoamine oxidase B; PAH, phenylalanine hydroxylase; TH, tyrosine hydroxylase.

TH is organized as a homotetramer with each subunit containing a regulatory domain (RD) that starts with a flexible N-terminus of varying length in the four isoforms, a central catalytic domain (CD) containing the non-heme iron as well as binding sites for substrate and cofactor, and an oligomerization domain (OD) that facilitates dimerization and subsequent tetramerization. This is the general subunit organization and oligomeric structure of all the mammalian AAAHs of which PAH is the only one where full-length structures of high resolution are available [74,75,76]. For TH, there is an NMR-structure of an RD dimer from rat [77] and crystal structures of CD + OD tetramers from rat [78,79] and human, and the details on how the RD interacts with the other domains is still uncertain (Figure 2). Most of the RD is an ACT-domain, a βαββαβ fold that is present in several proteins and serves a regulatory function, often by binding amino acids on the interface when they dimerize [80]. The TH-CD is described as a basket composed of helices and loops where the active site containing the active site iron is located in a 17 Å deep cleft at the center. The tetrameric organization is attained by dimerization of the CDs from two subunits mostly via H-bonds to each other, followed by tetramerization where the four 24-residue α-helices make a coiled-coil that is the only contact point between two dimers [78] (Figure 2). The presence of flexible hinges between domains and the long flexible N-terminus is most likely the reason why a high-resolution structure of full-length TH has been difficult to obtain, but it is highly valuable for the more precise prediction of potential effects of THD mutations. Due to the structural homology within the AAAHs, the effect of mutations causing TH enzyme defect (Figure 2) may also be predicted on the PAH structure by analogy from the multiple mutations in PAH observed in patients with phenylketonuria (PKU) [81,82]. There are 59 THD mutations that are deposited in the publicly available PNDdb (www.biopku.org, accessed on 15 October 2021). These include five nonsense mutations of which four lead to a TH truncated before the catalytic site, and 50 missense mutations (Figure 3). Actually, 39 (78%) of these missense mutations are at conserved sites in PAH, and 18 (36%) have identical residue substitutions in PKU as evaluated in the BIOPKUdb (www.biopku.org, accessed on 15 October 2021). As PKU patients usually present with a more well-defined biochemical phenotype based on plasma L-Phe levels [44] than THD patients where that depends on CSF levels of neurotransmitter metabolites 5HIAA, HVA, and MHPG as well as the HVA:5HIAA ratio, that can all display significant individual differences (for reference values, see [20]), comparisons with mutant PAH/PKU can contribute to the prediction of expected mutant TH/THD phenotypes.

Interestingly, S19C is the only mutation that has been reported in the RD. This mutation will abolish a phosphorylation site (see later) and has only been found in one patient which is in fact one of the few symptomatic carriers that are reported [83].

#### 1.5.4. Regulation of TH Activity

The regulation of TH is complex and tightly controlled on multiple levels. Understanding these processes can inform future strategies for treating disorders where TH function is compromised.

The medium- to long-term regulation of TH is executed on the gene expression level and involves transcriptional control, alternative RNA splicing, modulation of RNA stability, translational and post-translational regulation [84,85,86,87], while the primary modes of transient regulation affecting TH activity, location and stability occur through substrate availability [88,89,90], feedback inhibition by CAs [91,92], phosphorylation-mediated regulation [93,94], redox modifications, and protein–protein interactions.

Although the four human isoforms of TH display similar catalytic properties [72,87], their regulation may differ—potentially adding another layer of complexity to the control of CA biosynthesis, and possibly providing an avenue for differential, tissue-specific regulation to fulfil local demands [95]. As the presence and relative amount of each isoform varies among regions and may change between different stimulations and conditions [71], further studies are needed to determine the concrete physiological significance of these patterns of expression.

Signal-mediated phosphorylation of TH regulates its function in several ways (reviewed by Dunkley and Dickson [96]). S40 is the main site for TH activation, as its phosphorylation abrogates the binding of inhibitory CAs [97,98,99,100] and elevates the affinity for the pterin cofactor [101]. Feedback inhibition by CAs is considered an important regulatory mechanism on TH activity but also on stability [102]. Phosphorylation of S40 decreases the affinity of CAs 100-fold, largely through increased rate of dissociation, allowing reactivation of TH for catalysis [103,104]. The regulatory cross-communication between CA binding and S40 phosphorylation requires interactions involving not only the catalytic site (where the catechol-moiety coordinates to Fe^3+^), but also N-terminal residues [89,105,106]. The S40 site is targeted by several kinases [96], from which PKA-mediated S40 phosphorylation is best described. This kinase is also modulated by extracellular dopamine through inhibitory dopamine receptor 2 (D2R) auto-receptors on the pre-synaptic terminals and somatodendritic region, where they contribute to regulate TH activity, dopamine secretion and neuronal activity [107,108].

Although direct effects on TH activity have not been detected from phosphorylation on S8 and S19 [93], S19 is required for the high-affinity binding of regulatory 14-3-3 proteins [109,110], which are abundantly expressed in the brain and involved in the regulation of many cellular processes [111]. The activating 14-3-3 proteins were the first protein interaction partners identified for TH and the first identified function of 14-3-3 proteins [112]. However, considerable data are accumulating that show that TH is involved in several protein complexes including with protein phosphatase 2A (PP2A), AADC, GTPCHI, vesicular monoamine transporter 2 (VMAT2), α-synuclein, Hsc70, DJ-1, DnaJC12, and 5′-nucleotidase domain-containing protein 2 (NT5DC2) [106,113,114,115,116,117,118,119]. Several of these interactions may be relevant in THD and PD disease progression, and further investigations are warranted. Phosphorylation of TH has been linked to its cellular localization. For example, S31 phosphorylated TH immunoprecipitated together with VMAT2 and a-synuclein, and microtubular integrity was found to be important for the localization of TH to neurite projections [115], suggesting a role for S31 in transport of TH protein towards the terminal compartment. Hsc70 has also been found to interact with TH at presynaptic vesicles and might be a component in this vesicular protein-protein complex involving TH [118]. For several of the TH binding partners, the functional implication of the interaction is not well understood, such as for NT5DC2, which was recently found to play a role in Ser40 dephosphorylation of TH, but through a yet unknown mechanism [116]. It is likely that DnaJC12, a co-chaperone of Hsp70, is involved in the regulation of TH proteostasis as reported for PAH [119,120]. DJ-1 (or PARK7) has been found to activate TH and AADC through protection from redox stress [114] but also to regulate TH expression [121].

In summary, because TH fills such a central role as the rate-limiting enzyme in CA biosynthesis, precise homeostatic as well as adaptive regulation of its expression, turnover and activity is of critical importance for physiological functions that rely on dopaminergic neurotransmission. Hence, it should not be entirely surprising to see a regulatory domain that is largely devoid of mutations of pathological consequence. As most disease-associated variants disrupt either the structural integrity of the protein and/or hamper its catalytic performance, one could speculate on whether such loss of integrity may be to some extent compensated for by processes related to turnover, protein-protein interactions, or other molecular processes that are yet to be elucidated. Such theoretical considerations are especially pertinent since mutational severity on the protein level is often not reflected in clinical outcomes.

## 2. Current Treatment Options for THD

The mainstay of treatment for DRD has traditionally been the administration of L-DOPA, a treatment that is based on experiments done by Carlsson and colleagues in the late 1950s. In their studies they used rabbits treated with reserpine, a VMAT blocker that depletes brain dopamine and leaves the animals almost immobile and deeply sedated. Upon injection with L-DOPA, the animals were instantaneously restored to full mobility and wakefulness, providing the first evidence of not only the important role of dopamine in motor functions but also of the brain being able to metabolize L-DOPA to dopamine in the absence of vesicular dopamine loading and precise exocytotic release [122,123,124,125]. Later studies showed that AADC, which is localized in dopaminergic neurons, serotonin neurons and glial cells, is responsible for synthesizing dopamine from administered L-DOPA [29].

Currently, THD patients are mainly given L-DOPA in combination with an AADC inhibitor (carbidopa) that prevents its peripheral metabolism (Figure 3). Although resupplying L-DOPA improves the symptoms in some of the THD patients, it only partially alleviates symptoms or has little to no effect in others [126,127,128,129]. The patients responding well to L-DOPA treatment are generally categorized as having a mild form of THD, while the patients with little to no response suffer from the severe/very severe forms [20,126]. Of note, the use of L-DOPA for individuals belonging to the latter patient group can be limited by intolerable dyskinesias, even at low doses [129]. Therefore, the recommended starting dose of L-DOPA is low, and treatment must be tailored to the individual patient. For the patients with severe THD in particular, initial doses should be kept below 0.5 mg/kg daily and administered in multiple doses [2]. L-DOPA-induced dyskinesia can be amenable to amantadine, originally an influenza drug with unclear mechanism of action that is believed to work partly by inhibiting the re-uptake of dopamine [27].

Another drug, selegiline, has also been used to treat THD patients, usually in conjunction with L-DOPA/carbidopa. Selegiline is a selective monoamine oxidase type B (MAO-B) inhibitor, thus slowing down the catabolism of dopamine, and has improved symptoms in some THD patients [129,130,131,132,133]. The dopamine agonists bromocriptine and pramipexole have also been used together with L-DOPA, with limited additional effects [20]. A small number of patients have also been given synthetic acetylcholine antagonists such as biperiden and trihexyphenidyl with moderate success [133,134].

It is important to note that for BH4-deficiency related disorders several treatment options have recently become available, such as sapropterin dihydrochloride (Kuvan™) to restore BH4 levels. Kuvan™ is also used to treat BH4-responsive PKU. There are some indications that this currently available therapeutic may also be relevant for some THD patients as stabilizing effect of BH4 on THD associated mutants such as L236P, both shown as increased thermal stabilization of isolated TH1-L205P protein and as increased dopamine synthesis in in vitro transcription translation systems, has been shown [135]. Despite the fact that BH4 does not efficiently cross the BBB, oral supplementation with BH4 at high—though non-toxic—doses (100 mg/kg/day), resulted in significant increases in TH protein and activity in mouse brain extracts [135]. Kuvan™ treatment is, however, costly, and it is likely that a more efficient formulation for delivery of BH4 to the desired neuronal populations of the brain could improve this treatment option. In this regard, functionalizing gold nanoparticles with L-DOPA could improve BBB penetration by targeting the LAT-1 transporter [136]. Additional treatment for BH4-deficiency-related disorders typically includes inhibitors of monoamine metabolism and different targeting of the serotonin system, which becomes particularly compromised during L-DOPA treatment (see Opladen et al., 2020 [137] for more details).

Thus, the current therapeutic interventions used for THD are based on bypassing the TH deficiency by treatment with L-DOPA, dopamine agonists or inhibitors of dopamine metabolism (Figure 3) and can lead to the complete resolution of symptoms in some patients and significantly improve the quality of life in others. However, to date, a portion of patients still respond poorly or not at all to available treatments. Below we discuss ongoing efforts to develop novel therapeutics for THD, including therapies specifically directed at the underlying cause of THD, as opposed to the current regime of attempting to treat the disorder symptomology.

## 3. Promising and Future Treatment Opportunities for THD

The underlying cause of THD is *TH* mutations that generally affect the structure (misfolding/stability) and function (loss of activity and loss of interactions) of the enzyme leading to a loss-of-function phenotype [18]. Perhaps the most intuitive therapeutic strategy for THD is to develop an activator or to upregulate the levels of mRNA, protein, or activity of TH. This is challenging as the drug development industry has far more traction in gain-of-function diseases, where small molecule inhibitors and degraders can be used to reduce mRNA, protein, or activity levels [138]. However, there is currently an increasing number of emerging approaches—including the use of enzyme replacement therapy, pharmacological chaperones, and gene therapy—that in theory can be adapted for specific loss-of-function disorders. Below, we discuss ongoing efforts to apply some of these approaches for the development of novel therapeutics for THD.

### 3.1. Enzyme Replacement Therapy

As described above, THD is a disorder that arises from mutations in a key enzyme of a major metabolic pathway. One promising approach to treat such disorders is the administration of the wild-type enzyme to compensate for the low activity of the impaired one using enzyme replacement therapy (ERT) [139]. In ERT it is crucial to deliver the enzyme to the specific deficient tissue and cell type. In the case of THD and other disorders with deficient TH, such as PD [140] and some neuropsychiatric disorders [141,142], this would entail delivering TH across the blood brain barrier (BBB) to induce production of L-DOPA in situ in the brain. Recombinant TH as an apoenzyme is prone to lose its activity over time at 37 °C in vitro [143], thus finding ways to increase wild-type enzyme delivery across the BBB, yet preserving enzymatic stability and activity, has been challenging. However, promising advances towards this aim have been made in recent years by using appropriate nanoparticle (NP) carriers to deliver TH across the BBB [144]. Relevantly, Bezem et al. recently optimized TH expression and purification to yield enzyme preparations with improved stability and activity [143]. In combination with porous maltodextrin NP, they used these enzyme preparations to bind, stabilize, and deliver TH to neuronal cells, both in cell culture and in mouse brain [145]. Crucially, the stability of TH was maintained through loading and preserved during storage, and there was an increase in intracellular L-DOPA synthesis following NP uptake [145]. The same group also investigated the interactions between TH and a more modifiable and biodegradable NP, namely, porous silicon nanoparticles (pSiNPs) [146]. This study showed that the enzymatic activity of TH remained intact upon binding to the pSiNP surface, and pSiNPs could represent another promising device for therapeutic delivery of TH [146]. This could potentially be achieved through intravenous injection, which would require pSiNP-bound TH to be stabilized during circulation followed by uptake into the brain by crossing the BBB. Another possible approach is by direct delivery to the brain using hydrogel implants containing TH-loaded pSiNPs [146]. However, it is important to note that the use of ERT for THD has not yet been tested in live animals, and this approach must be further investigated and optimized in future work.

### 3.2. Pharmacological Chaperones

Cells have evolved numerous quality control mechanisms to protect against aberrant cellular activity, and proteins that are recognized as improperly folded are generally targeted for proteasomal degradation. Proteins with mutations that cause relatively modest changes in conformation and that do not compromise functional integrity may still be recognized as misfolded and hence be prematurely degraded, leading to a loss-of-function phenotype [147]. A number of the THD-associated mutations have been linked to TH instability and loss-of-function misfolding [18,69,148,149], potentially causing rapid degradation of mutant protein resulting in the decreased TH and dopamine levels found in THD patients [149]. Finding small molecular weight compounds with pharmacological chaperone (PC) potential able to stabilize TH protein and enzymatic activity in vivo therefore appears relevant for THD. The use of PCs is also being investigated to treat other genetic disorders where protein misfolding and instability are central pathologies. Examples include PKU [150], Friedreich’s ataxia [151] and most notably lysosomal storage disorders, for which the chaperone drug migalastat has already been approved for Fabry disease and a number of other PCs are currently in clinical trials [152,153,154].

A number of potential PCs have already been discovered for TH, and their effects on the enzyme have been investigated in vitro, in cells, and in animal models [149]. The search for PCs commonly includes investigating derivatizations of natural ligands, such as cofactors, and in the case of TH the cofactor BH4 has been reported to have chaperone effect for TH [135]. However, the mechanism remains unclear, and other studies have found that BH4 can cause TH aggregation [155]. Another approach for TH has been to study compounds previously identified as having PC-effect for PAH-and PKU-associated mutants [156]. For example, in one study, four such compounds were investigated for chaperone effect for TH and TPH2 [70]. Oral administration of one of the compounds, compound III, to wild-type mice increased the total TH activity in mouse brain extract by 100%, with no effect on TPH activity or mouse dopamine levels. In addition, this compound stabilized the human THD associated mutant R233H (TH1-R202H) in vitro, proving for the first time PC as potential treatment of THD and other disorders associated with TH misfolding [70].

In search of PCs more specific for TH, the same group performed high-throughput screening based on differential scanning fluorimetry of 10,000 compounds [149]. Several hits bound to TH, and out of 12 that significantly protected wild-type hTH1 and mutant R202H from time-dependent loss of activity, three were selected for further detailed characterization. Two of these hits (named Compounds **2** and **4**) had a clear stabilizing effect, while the last one (named Compound **5**) showed low conformational stabilization of hTH1 but was still able to protect the enzyme activity to a high extent. This compound was able to increase the activity for not only hTH1, but also the THD-associated mutants p.TH1-L205P, p.TH1-R202H and p.TH1-Q381K, indicating that protection from inactivation by some PCs is not only from conformational stabilization of TH. Compounds **4** and **5** had weak competitive binding to the BH4 binding site. Using electron paramagnetic resonance and molecular docking analysis the mechanism behind their TH activity protection was found to be from interaction with the active site iron. Thus, the novel modes of activity protection detected in these potential TH PCs suggest that combinations of chemically optimized compounds with different mechanisms of action is a promising strategy for THD drug development [149]. More recently, 1280 approved small molecule approved drugs were screened for TH PC potential and one of the hits was levalbuterol, the active molecule in salbutamol/albuterol [157]. This drug is a bronchodilator widely administered for chronic obstructive lung disease and asthma and has been linked to reduced risk and delayed progression of PD and other neuropsychiatric and neurodegenerative disorders [158,159,160,161,162]. Of interest, salbutamol is also suggested to improve the response to L-DOPA therapy in PD by enhancing L-DOPA transport across the BBB [163,164,165]. When investigating the in vitro effects of levalbuterol on TH, dopamine was included for comparison as it is the most characterized natural stabilizer of the enzyme. Levalbuterol was found to bind and stabilize TH with reduced affinity compared to dopamine, but without affecting TH activity [157]. Furthermore, as dysregulated TH has a propensity to self-oligomerize and make large aggregates when interacting with membranes in absence of its protein partners [166], the effect on TH aggregation was also tested. Interestingly, both dopamine and to a lesser extent levalbuterol delayed the formation of large TH aggregates [157]. This report serves not only as an example of how previously approved drugs may potentially be repurposed for new applications, but also of how such studies may reveal previously unknown regulatory mechanisms such as the anti-aggregating effect of dopamine on TH.

### 3.3. Gene Therapy

Reversal of causative *TH* mutations through genetic manipulation is theoretically possible using gene editing. The newly developed CRISPR-Cas9 genome editing technologies are promising tools for in vivo base correction [167,168,169] and have been used to rescue the disease phenotype in a mouse model of PKU by changing a single base [170]. More recently, evidence of CRISPR-Cas9-based in vivo gene editing in humans has been published [171]. The interim data from an ongoing phase I clinical study (#NCT04601051) on hereditary transthyretin (hATTR) amyloidosis, a progressive fatal disease with an autosomal pattern of inheritance and tissue accumulation of misfolded transthyretin (TTR) protein, shows encouraging results [171]. Intravenous infusion with the therapeutic NTL-2001, which consists of a lipid NP delivery system with liver tropism intended to edit TTR in hepatocytes, resulted in only mild adverse events and led to decreases in serum TTR protein concentrations in all hATTR patients enrolled in the study [171]. As for ERT and PC described above, the use of gene therapy for THD patients would require delivery of the therapeutic agent to the brain. This would usually entail a delivery system able to cross the BBB, however, recent research efforts into utilizing gene therapy for inherited neurodegenerative disorders have used a different approach [172,173]. For example, in a mouse model of neuropathic Gaucher disease, caused by mutations in the *GBA* gene leading to deficient glucocerebrosidase protein, an adeno-associated virus vector was administered by fetal intercranial injection [173]. This resulted in reconstituted glucocerebrosidase expression, and the mice were viable up to at least 18 weeks, fully mobile and fertile [173]. Such delivery to the developing CNS may be especially important in some patients with severe THD, where pathology is already present at birth. The use of fetal gene therapy would rely on accurate prenatal diagnosis, usually limited to families with a history of severe THD in older siblings. As described above, only two instances of prenatal diagnosis of THD have been reported in literature so far, both with genomic DNA extracted from the chorion-villus [25,26]. Future research efforts will hopefully elucidate how this approach can be adapted for THD patients.

## 4. What Is Needed for a Personalized Medicine Approach in DRD?

There are some infrastructures that need to be in place before the PM approach can be exploited: (1) tailored diagnostic and clinical protocols and (2) an expanded repertoire of treatment options. Both of these depend on each other. From a diagnostic point of view, it is of little value to know the exact genotype and expected phenotype of patients if they are not reflected in different treatment options. There are however promising treatments in the pipeline for many rare disorders, including DRD, that will stimulate a change in the clinical practice in the future. A PM approach will require a much more accurate genotype-phenotype mapping. This sets high demands on prediction tools that can be used in the clinical evaluation of patients. Recent developments within computational approaches will be a large part of predictive approaches. However, we argue that biochemical and cellular characterization of mutations will be a highly valuable data resource that can be combined with systems medicine modeling to improve prediction needed for clinical evaluation of the individual patient. We illustrate this for the case with THD patients but will be similar for other DRD-related enzyme deficiencies. Below we describe: (I) biochemical and cellular assays to characterize existing and new mutations to generate a data resource of sufficient quality and richness and (II) how systems biology models can be used to transform data resources into patient specific models that can be useful in the clinical evaluation and treatment of individual THD patients.

### 4.1. Generating Relevant Data about Possible THD Mutations

#### 4.1.1. Biochemical Assays Using Purified Proteins

As TH is mainly expressed in the adrenal glands and nervous system, it is not feasible to obtain mutant enzyme from patients. Thus, an alternative is to characterize THD-associated mutants in vitro as recombinantly expressed and purified enzymes. In 1991, before the first THD-associated mutant was discovered, Le Bourdelles et al. [68] cloned TH into a bacterial expression vector to enable production of pure preparations of TH1 and TH2 that were unphosphorylated and without bound catecholamines. As previously discussed, structural characterization of TH using conventional methods has been hindered by the dynamic nature of the enzyme. However, several competing approaches were developed for assaying recombinant TH specific activity [174,175,176].

Today, biochemical data using TH1 is available for more than 30 THD-associated variants [18,69,70,148,177]. These include the first two reported mutations, Q412K (TH1-Q381K) and L236P (TH1-L205P), respectively, that were characterized in pure form upon reporting or immediately after [148,177]. These early patients were homozygous: two brothers with Q412K and a girl with L236P, where the more severe phenotype of the latter corresponded well with the biochemical data that revealed the L236P variant retained almost no residual activity [148]. However, it has become clear since that results derived from in vitro assays are often not indicative of the severity of the overall clinical picture in the affected patients and vice versa. In 2005, Royo et al. characterized four novel mutations (i.e., T245P, T283M, R306H, and T463M) that were identified in three male patients from two families, all carrying the disease-causing alleles in a heterozygous fashion [69,178]. Common for the patients was an almost complete remission of symptoms with low-dose L-DOPA treatment and no side-effects even after more than 20 years. A likely explanation for the mild phenotype was that despite the fact that all four mutants presented reduced stability that is expected to cause reduced TH protein levels in vivo, the catalytic function of the existent protein was not affected [69].

By far the largest study of THD mutants is that by Fossbakk et al. who expressed and purified 23 THD-associated variants and characterized their effects on solubility, stability, activity and substrate specificity [18]. In this work, a correlation was found between mutations linked with low residual activity and type B patient classification. The dominant feature of the most common THD mutation R233H (TH1-R202H), that was found in both heterozygous and homozygous allele combinations in type A as well as B patients [20] is low residual activity [18,70]. Furthermore, an exhaustive study including isolated TH1-R202H protein and investigations in homozygous *Th* knock-in mice with the equivalent mutation Th-p.R203H showed the reduced TH (protein and activity) and dopamine levels in vivo [179]. This study also revealed that the mutation may hamper TH transport and proper localization in the brain striatum. Analyses of a unique brain from a 16 week old miscarried human fetus affected by the severe form of THD with R328W-T399M *TH* genotype have revealed a large decrease in TH levels but also in VMAT1, VMAT2, and D2R, and in the developmental of markers for synapses, axons, and dendrites, providing information on the role of dopamine in neurodevelopment [180].

As it was recently reported, the tetrameric assembly of TH itself may become compromised by mutations and intriguingly, in the case of one mutant where residues within the oligomerization domain are affected (D467G), a partially active dimeric form—that may be transient in nature—was detected [181].

Taken together, in vitro biochemical investigations revolving around the kinetic/mechanistic consequences of known mutations serve an important role in basic research and is expected to benefit efforts to develop novel, targeted PCs aimed at providing more tailored treatment for THD. The structure of the full-length enzyme has now been finally solved thanks to advances in cryoEM [105]. It can be anticipated that this structural information will yield further meaningful contributions to the understanding of disease mechanisms at the molecular level as well as in the screening of compounds for stabilizing effects that may rescue lost enzyme function (see in Section 3).

#### 4.1.2. Cell Assays to Assess Changes in Proteostasis

Cell lines give the opportunity to study proteins in a more physiological context than isolated proteins, while still representing a simpler and more controllable environment than animal models. Expression of TH protein in cell cultures is first and foremost a simple way to study the effect of mutations on steady-state protein levels, which reflects the stability of the protein. This has for example been done for the THD associated mutants Q412K (TH1-Q381K), R233H (TH1-R202H), and L236P (TH1-L205P) in an adherent PC12 cell line, where the protein levels of all three mutant forms were only ≤25% of that of wild-type [149]. TH activity in cell lysates was also 5–15% of that measured from cells expressing wild-type TH [149], which correlates well with reported in vitro solubility and thermal stability of the mutant proteins [18]. Since many cases of THD are caused by heterozygous TH mutations, co-expression studies could potentially improve the understanding of genotype–phenotype correlations. Co-expression of PAH mutants has been studied in COS-1 cells, where no interallelic effect as well as negative interallelic complementation (combination gives lower enzyme activity than predicted) have been reported [182,183]. In vitro experiments with heterotetramers of wild-type and N-terminally truncated hPAH (ΔN102-hPAH) or two mutant alleles have also shown evidence of negative interallelic complementation [184,185].

Novel treatment options aiming to stabilize misfolded mutant proteins are often first identified by in vitro screening assays. The effect also needs to be demonstrated in cell cultures, where fewer of the stabilizing compounds tend to demonstrate increased steady-state protein levels and/or enzyme activity due to issues such as compounds not binding target protein that is involved in protein interactions, off-target binding, inability to cross the plasma membrane or cytotoxicity. As presented above (Section 3.2) cell culture studies (PC12Adh) with compounds that are in vitro stabilizers of TH have demonstrated examples both of canonical PCs increasing both TH protein level and activity, compounds only protecting enzyme activity, as well as compounds failing due to cytotoxicity [149]. Mutation specific effects of compounds were also reported in this study [149].

Besides studying steady-state protein levels, immunostaining and fluorescent microscopy can provide valuable information about subcellular localization and co-localization with relevant molecular markers. This has recently been studied in liver tissue from mice expressing the PKU-associated PAH-R261Q mutant. PAH-R261Q in liver tissue showed increased ubiquitination compared to wild-type PAH and was also present in amyloid-like aggregates co-localizing with autophagy markers [48]. The discovery of mutant PAH aggregates together with oxidative stress in mice establish that PKU can also be a gain-of-function disease [48]. Whereas studies in animals allow for this correlation between markers and physiological processes, additional mechanistic data can be gained from cell culture. For example, a more thorough insight in the autosomal degradation of aggregates may be achieved by applying activators or inhibitors of autophagy or the proteasomal degradation pathway. Inhibition of proteasomal degradation in PC12D cells leads to ubiquitin-positive insoluble aggregates of Ser-40 phosphorylated TH [186] and could compromise lipid bilayer integrity and thus cell viability over time [166]. Together, these works highlight the importance of understanding disease mechanisms also at a molecular and cellular level, and the benefit of treatment options directly targeting unstable protein structures.

#### 4.1.3. Complex Multi-Cell Cultures and Patient Derived iPSC

Several novel ways to model human biology and disorders in vitro are emerging. One such example is the organs-on-chips (Organ chip) system [187]. This system was recently used to develop an in vitro human BBB model able to recapitulate high level barrier function for over a week in culture. This was achieved by designing a 2-channel microfluidic chip lined by induced pluripotent stem cell (iPSC)-derived [188,189] human brain microvascular endothelium interfaced with human brain astrocytes and pericytes [190]. The resulting «BBB Chip» could represent a preclinical drug development tool for the validation of delivery systems that transport brain-targeted therapeutics across the human BBB [190] and could therefore be relevant for the future development of therapeutics for THD. Theoretically, such a system could also be used to investigate individual THD patient pathophysiology in vitro by using iPSCs generated from patient fibroblasts, and the first such line (TH-1 iPSC) has already been generated from a male patient with TH compound heterozygous variants (R129X, R231P) [191]. Additionally, the use of iPSC as cell replacement therapy is already being investigated in a clinical trial for the treatment of PD [192] and, if successful, this approach could also benefit patients with THD. Thus, novel systems such as the “BBB chip” have the potential to become important tools for PM, used for tailored pathophysiological studies of THD and for drug testing, while iPSC technology has the added potential of possibly being used directly in treatment of THD patients.

### 4.2. Computational Modeling for a Personalized Medicine Approach in THD

For metabolic disorders, a large part of the genotype—phenotype mapping can be made if one can quantify the extent to which alterations in enzymatic properties and their regulatory circuits affect the metabolic pathways in which they participate. Such a mapping would typically be represented by a mathematical model of the metabolic pathway(s) of interest. This type of modeling is common in systems biology, and a range of modeling approaches are available with different complexity and explanatory power. The flux balance type of analysis is a coarse modeling approach that is frequently used, where only the stoichiometries of the reactions are considered. This approach is typically used for large, often genome-wide, metabolic models [193].

More comprehensive approaches include Michaelis-Menten type kinetic representations of the enzymes, that would typically include allosteric interactions, substrate competition, and impact of signaling mediated regulation of the enzymes [194]. Such models would rely on detailed kinetic characterization of the enzymes, which however is only rarely available at a satisfactory detail. Still, a range of different compromises and hybrid approaches can be used to build models according to the available knowledge basis [195]. An interesting feature of such models is that they in principle can easily implement individual differences and environmental factors that are known. This could be individual differences in protein expression or turnover (V_max_ values for enzymes), binding affinities (e.g., from missense variants, polymorphisms or different isoform expression), differences in circulating metabolite levels, transport and tissue consumption, signal-mediated regulation (e.g., via hormones), or drug pharmacokinetics.

#### 4.2.1. Systems Modeling of DA Synthesis and Metabolism

Dopamine synthesis and metabolism has been modeled in several studies (Table 1), addressing different regulatory aspects and pathological mechanisms. The majority of models have used the Michaelis–Menten type of kinetics with the exceptions of the modeling studies from the Voit lab (which uses the power law formalism) [196,197] and notably the hybrid modeling approach by Nekka and coworkers [198]. The latter combines a two-compartment model of L-DOPA pharmacokinetics, a simplified model of extracellular dopamine and a neurocomputational model of the basal ganglia. This approach could be extended with more detailed model modules, that would also implement individual variation and disease specific features (more below). In the more comprehensive models of DA synthesis and metabolism, the temporal changes in metabolite concentration can be followed in response to changes in levels of precursors amino acids, enzyme expression, alterations in enzyme states (e.g., post translational modifications, PPIs) or parameter values (such as K_m_, K_i_, and k_cat_). This is illustrated by Nijhout et al. [199], where extracellular dopamine levels were modeled for different combinations of polymorphisms in TH and DAT (Table 1). Although Best et al. (2009) and Reed et al. (2012) (Table 1) incorporate regulation by TH Ser40 phosphorylation and autoreceptor feedback regulation, they did not explicitly model different states of TH such as done Kaushik et al. [200], where the phosphorylation and dephosphorylation reactions and redox transitions were included.

The main objective of the models available in the literature is typically to dissect regulatory mechanisms and the impact of molecular properties on systems features such as flux control, conservation of homeostasis or robustness/tolerance. They are not primarily designed to predict the presence of pathology but can make predictions about altered pathway phenotypes provided sufficient biochemical measurements are available. Generally, models that describe DA synthesis and metabolism in detail, do not include neuronal activity or post-synaptic signaling. There are, however, other studies that focus on understanding post-synaptic dopamine signaling [201,202] and more extensive neurocomputational models of basal ganglia connectivity and function [203,204]. A feature that is lacking from current models of presynaptic DA homeostasis is the synthesis and homeostatic regulation of BH4. This would be a useful extension of the models for increased relevance for patients with BH4-deficiency, but also THD. Homeostasis is frequently used when discussing metabolic disorders, but also neurotransmitter disorders. Concepts and approaches from control engineering could be useful for analyzing the systems of interest with respect to conditions that are required for homeostasis and when breakdown can be expected [205].

**Table 1:** A selection of interesting mathematical modeling studies of catecholamine synthesis and metabolism.

Study	Model Type	Pathways	Objectives	Comments
Justice et al., 1988 [206]	ODE, MM	DA synthesis, release, metabolism	First of its kind—initial computational study	Compartmentalization of cytosolic DA
Kaushik et al., 2007 [200]	ODE, MM	DA synthesis, TH regulation by S40 phosphorylation/BH4 levels/Fe oxidation	Impact of TH regulation on DA levels	Focused on TH regulation, PKA phosphorylation, dephosphorylation and impact of α-Syn on dephosphorylation
Qi et al., 2008 [196,197]	ODE, PL	DA synthesis and metabolism	Analysis of presynaptic DA homeostasis	Detailed on DA metabolites, catecholamine auto-oxidation, melanin formation
Best et al., 2009 [207]	ODE, MM	DA synthesis, metabolism and release	Analysis of homeo-static mechanisms in DA synthesis and release	Models effect of substrate inhibition on DA homeostasis. Models regulation of TH by auto-receptors
Reed et al., 2012 [208]	ODE, MM	DA and serotonin synthesis and metabolism	Modeling the impact of L-DOPA treatment	Models DA synthesis in serotonergic neurons during L-DOPA treatment
Nijhout et al., 2014 [199]	ODE, MM	Several are discussed including DA synthesis and metabolism	Models impact of protein variants on DA homeostasis	Discusses homeostasis and robustness and illustrates system tolerance, e.g., towards enzyme variants of TH and DAT that have altered V_max_
Cullen & Wong-Lin 2015 [209]	ODE	Reduced model of Best et al., 2009	Increase computational efficiency	Similar predictions as Best et al., 2009. Less intuitive to implement alterations in kinetic parameters
Véronneau-Veilleux et al., 2021 [198]	ODE	Pharmacokinetic model of levodopa, synaptic DA, and impact of DA on basal ganglia circuit activity	Models L-DOPA treatment in Parkinson’s disease	Hybrid model of three different modeling approaches illustrates how different models can be combined to bridge interactions between different subsystems and obtain clinically relevant predictions

MM, Michaelis Menten kinetics; ODE, ordinary differential equations; PL, power law formalism as in Biochemical Systems Theory.

#### 4.2.2. Implementing Systems Medicine in Personalized Medicine for DRD

Systems medicine involves the application of systems biology to medicine, but also naturally integrates other disciplines and is geared towards a PM perspective [210]. Data rich resources will become important tools to tackle also more complex metabolic disorders and to understand individual differences. The Recon3D resource is presently the most comprehensive human metabolic network model, where metabolic reactions are mapped to genes on genome scale and linked to three-dimensional structures of metabolites and proteins [211].

One could claim that simple monogenic disorders of metabolism are well enough understood without considering quantitative aspects of the pathways of which the enzymes are functioning. Biochemical and cellular models will provide background information about changes in enzyme kinetic properties and protein turnover by different genetic mutations (see Section 4.1 above). Still, unless there is a very dramatic effect, such as loss of function, there is usually not a clear-cut relation between the protein perturbation and its effect on the total pathway behavior. In addition, for these rare disorders, the majority of patients carry two different mutations, and their combined effect is not easy to evaluate. Mathematical models can, however, be applied in such cases, provided there is available biochemical data on the mutations. The biosynthesis pathway models will provide estimates on the expected perturbation at the pathway level. An important prerequisite for this is quality data resources. Pathway modeling results are expected to be well correlated with the metabolite phenotype measured in CSF or serum, in particularly if exchange rates between blood, CSF and brain tissue are known for precursor and monoamine metabolites. However, a pathway model of monoamine synthesis and metabolism will, similarly as CSF or serum metabolite profiles, not necessarily give a clear answer about the disease severity for individual patients.

Another useful application of systems biology models is to perform in silico testing of different treatment strategies. Thus, different combinations of treatments, in addition to or replacing L-DOPA, can be tested for existing options; either dietary, precursor or cofactor supplementation, DA metabolism and reuptake inhibitors, off-target compensatory treatments (e.g., towards serotonin with precursor supplementation 5-OH-Trp or selective serotonin reuptake inhibitors); or in the future by novel treatment options, such as PCs (above). An interesting example of L-DOPA off-target modeling was published by Reed et al. (2012) [208], where the impact of L-DOPA treatment on DA synthesis in serotonergic terminals is modeled. Pharmacokinetic compartment models of different drugs can be coupled to the DA model to get temporal estimates of drug doses experienced in the target cells, e.g., striatal dopaminergic terminals. These models can be generated for different drugs and drug combinations and can be made “individualized” if pharmacokinetic or pharmacogenomics data are available for the patient and could be used to plan drug administration.

## 5. Concluding Remarks

**Figure 4:**
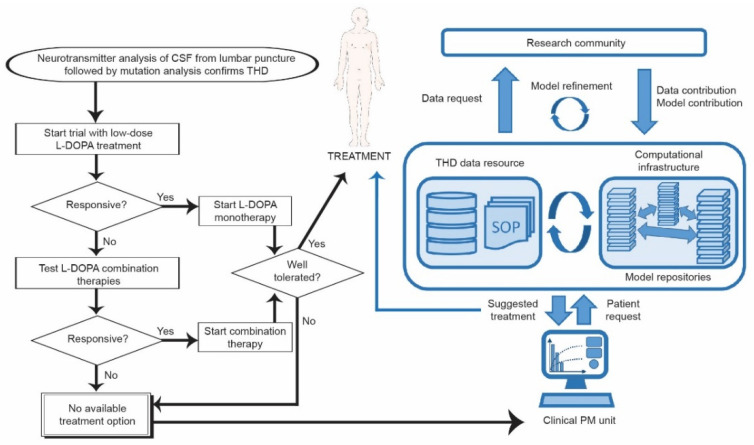
Towards personalized medicine approaches for THD. For both current and future treatment approaches the starting point is the diagnosis of THD from neurotransmitter analysis of CSF from lumbar puncture, followed by mutation analysis of the *TH* gene. The current mainstay treatment for THD is the administration of L-DOPA and carbidopa [2] (depicted as L-DOPA monotherapy in the figure). To date, some patients with unfavorable response to L-DOPA monotherapy have been given additional therapeutics, such as dopamine agonists or inhibitors of dopamine metabolism (see text for details). Biochemical and cellular assays have been used to study a number of *TH* mutations, and such assays are currently being used in research efforts to develop novel THD therapeutics. Such ongoing efforts may lead to PM approaches for THD (in blue). We expect that in the future, data from biochemical and cellular assays may be fed into computational modeling infrastructures to aid in the prediction and selection of the best available treatment and delivery options for individual THD patients. Abbreviations: CSF, cerebrospinal fluid; THD, tyrosine hydroxylase deficiency; L-DOPA, 3,4-dihydroxyphenylalanine; SOP, standard operating procedure; PM, personalized medicine.

Symptom variability seems to be a fundamental feature of THD, even occurring in siblings with identical mutations [20]. It is possible that this phenotypic plasticity is sensitive to multiple genetic and/or environmental modifiers that synergize or cancel out to drive or inhibit symptom development. The emergence of new “omics technologies” is likely to contribute to elucidate such complex disease mechanisms. We anticipate that these technologies, coupled with the ongoing paradigm shift towards PM described in this review, will benefit THD patients. For example, to be able to accurately predict the expected neurobiological consequences of TH mutations in individual patients, one emerging option is to integrate data from biochemical and cellular assays with systems modeling based on detailed knowledge on TH regulation and signaling (Figure 4). Systems modeling may also assist clinicians in the evaluation and design of different treatment options for the individual patient, given that a larger set of possible treatments is available. Computational approaches within machine learning are becoming very powerful and are likely to be an important component of PM at least for more common diseases.

There is currently no cure for THD, and while the majority of patients experience symptom improvement in response to L-DOPA, a significant percentage is currently without effective treatment options. New treatments are therefore needed, and this will likely have to rely on governmentally funded drug development. For THD, one benefit could be the more numerous disease-associated missense variants known for PAH, the “sister enzyme” of TH. PKU is a much more frequent disease, and lessons learned as well as candidate drugs being developed for PKU could also be possible candidates for TH mutants and benefit THD patients. Although there are different challenges with drug delivery to the brain and TH regulation is different and apparently more complex, a coordinated drug development strategy for the AAAHs could help to overcome some of the difficulties of funding drug development for rare diseases. In this regard, the International (https://irdirc.org/, accessed on 15 October 2021) and newly started European (https://www.ejprarediseases.org/, accessed on 15 October 21) consortiums for research on rare diseases are important platforms for increasing collaboration and development in the rare disease field.

For a future implementation of a PM approach, access to data resources and data sharing are of key importance. Such resources should be supervised, use standardized reporting formats and assessment protocols, and need to be run under long-term funding programs. A possible format for data generation would then be community-driven where expert laboratories on the protein(s) of interest contribute or dedicated infrastructures can perform the required characterization of existing and new disease mutations. It would also be natural that model repositories of certified models and best-practice computational procedures to be handled by international resources as well, possibly with local nodes. Bioinformatics infrastructures, such as ELIXIR in Europe, could be possible hosts for data and model repositories, and computational resources. Patient queries from different clinical specialist departments could then be handled in a sensitive way and evaluated—automatically or by communication between specialists at the resource- and clinical centers.

In conclusion, some challenges remain, but hopefully increased awareness about the complex THD phenotypes coupled with technological advances will result in timely correct diagnoses and better treatment options for patients suffering from THD in the future.

## Data Availability

Data sharing not applicable.

## Abbreviations

3-MT	3-methoxytyramine
5-HTP	5-hydroxytryptophan
5HIAA	5-hydroxyindolacetic acid
5HIAA	5-hydroxyindolacetic acid
AAAHs	aromatic amino acid hydroxylases
AADC	aromatic acid decarboxylase
BBB	blood–brain barrier
BH4	Tetrahydrobiopterin
CA	Catecholamines
CD	catalytic domain
CNS	central nervous system
COMT	catechol-O-methyltransferase
CSF	cerebrospinal fluid
D2R	Dopamine receptor 2
DBH	dopamine beta-hydroxylase
DOPAC	3,4-dihydroxyphenylacetic acid
DRD	dopa-responsive dystonia
ERT	enzyme replacement therapy
GTPCHI	GTP cyclohydrolase I
hATTR	hereditary transthyretin
HVA	homovanilic acid
L-DOPA	3,4-dihydroxyphenylalanine
LAT1	large neutral amino acid transporter
LNAAs	large neutral amino acids
MHPG	3-methoxy-4-hydroxyphenylethylene glycol
MN	Metanephrine
MAO-A	monoamine oxidase A
MAO-B	monoamine oxidase type B
MPHG	3-methoxy-4-hydroxyphenylethylene glycol
NMN	Normetanephrine
NP	Nanoparticle
NT5DC2	5′-nucleotidase domain-containing protein 2
OD	oligomerization domain
PAH	phenylalanine hydroxylase
PC	pharmacological chaperone
PD	Parkinson’s disorder
PKU	Phenylketonuria
PLP	pyridoxal phosphate
PM	personalized medicine
PNMT	phenylethanolamine N-methyltransferase
PP2A	protein phosphatase 2A
pSiNPs	porous silicon nanoparticles
PTPS	6-pyruvoyl tetrahydrobiopterin synthase
RD	regulatory domain
SOP	standard operating procedure
SR	sepiapterin reductase
TH	tyrosine hydroxylase
THD	tyrosine hydroxylase deficiency
TPH1	tryptophan hydroxylase 1
TPH2	tryptophan hydroxylase 2
TTR	Transthyretin
Tyr	Tyrosine
VMAT1	vesicular monoamine transporter 1
VMAT2	vesicular monoamine transporter 2
VTA	ventral tegmental area
WT	wild type
